# Editorial: Polysaccharides for health and nutrition - extraction, isolation, bioactivity and innovative applications

**DOI:** 10.3389/fnut.2026.1833026

**Published:** 2026-04-02

**Authors:** Anderson Junger Teodoro, Orquidea Vasconcelos dos Santos, Rita Carneiro Alves, Fozia Kamran, Barbara Elisabeth Teixeira-Costa

**Affiliations:** 1 Integrated Nutrition Center (CIAN), Department of Nutrition and Dietetics, Federal Fluminense University, Niterói, RJ, Brazil; 2 Graduate Program in Food and Nutrition (PPGAN), Federal University of the State of Rio de Janeiro (UNIRIO), Rio de Janeiro, RJ, Brazil; 3 Graduate Program in Food Science and Technology (PPGCTA), Federal University of Pará (UFPA), Belém, PA, Brazil; 4 Chemistry and Technology Network (REQUIMTE), Faculty of Pharmacy, University of Porto, Porto, Portugal; 5 School of Science, Western Sydney University, Penrith, NSW, Australia

**Keywords:** carbohydrates, dietary fibers, functional compounds, green extraction, microbiota modulation, nutrition, polysaccharides, prebiotics

## Introduction

Polysaccharides constitute one of the most structurally diverse classes of biomacromolecules found in nature and play essential roles in human nutrition and health. These complex carbohydrates, derived from plants, algae, fungi, bacteria, and agricultural biomass, are increasingly recognized for their multifunctional properties in both biological systems and food technologies. Recent research has demonstrated that polysaccharides participate in fundamental physiological processes including immune regulation, metabolic modulation, and gut microbiota interactions, highlighting their importance as dietary bioactive compounds and functional food ingredients.

The Research Topic “*Polysaccharides for Health and Nutrition—Extraction, Isolation, Bioactivity and Innovative Applications*” brings together a collection of studies addressing emerging advances in polysaccharide extraction, purification, structural characterization, and biological functionality. The articles collectively explore how polysaccharides derived from both conventional and unconventional sources can contribute to human health and how their biological activities are influenced by extraction methods, structural properties, and processing conditions.

One of the key themes emerging from the Research Topic concerns advances in extraction and purification technologies. Traditional extraction methods such as hot-water extraction remain widely used; however, several contributions highlight the advantages of modern techniques—including enzyme-assisted extraction, ultrasound-assisted extraction, microwave-assisted extraction, and using green solvent systems—to enhance yield, efficiency, and structural preservation of polysaccharides. For example, the review “*Advances in the Extraction, Purification, Structural Characterization and Biological Activities of Polysaccharides*” provides a comprehensive overview of recent progress in extraction technologies and structural analysis, emphasizing how optimized processing conditions can influence both molecular structure and functional properties of polysaccharides (Yu et al.).

In addition to extraction strategies, structural characterization represents another fundamental aspect addressed in this Research Topic. Polysaccharides exhibit remarkable structural complexity, including variations in monosaccharide composition, glycosidic linkages, branching patterns, and molecular weight distribution. These structural attributes strongly influence their physicochemical properties and biological functions. The article “*Isolation, purification, and characterization of Dendrobium polysaccharides*” highlights how differences in species origin, extraction methods, and structural features can affect antioxidant, anti-inflammatory, antimicrobial, and immunomodulatory activities of polysaccharides (1).

Another major focus of the Research Topic is the evaluation of the biological activities of dietary polysaccharides. Increasing evidence indicates that these macromolecules exhibit multiple bioactive properties, including antioxidant, anti-inflammatory, immunoregulatory, and metabolic regulatory effects. Such activities are often associated with the ability of polysaccharides to interact with immune receptors, modulate signaling pathways, and regulate oxidative stress. These findings reinforce the concept that polysaccharides are not merely structural components of foods but also function as bioactive molecules capable of contributing to disease prevention and health promotion.

The studies in this Research Topic explored the interaction between polysaccharides and gut microbiota. Non-digestible polysaccharides act as prebiotic substrates that selectively stimulate beneficial microbial populations in the gastrointestinal tract, through fermentation and the production of short-chain fatty acids and other metabolites. These compounds influence metabolic homeostasis, immune function, and intestinal barrier integrity. Key findings from “*Fractional ethanol precipitation modulates the structure and function of Coprinus comatus mycelial polysaccharides: alleviation of DSS-induced injury in Caco-2 cells via regulation of mitochondrial function*” show that optimization of fractional ethanol precipitation from mycelial polysaccharides can enhance its structure and mitigate DSS-induced Caco-2 cell injury through mitochondrial regulation (Chen et al.). While in, “*Advances in the extraction, purification, structural characterization, and elucidation of the biological functions of polysaccharides from Hericium erinaceus*,” the polysaccharides from this edible mushroom have relevant biological properties such as gut modulation, antitumor, antioxidant, hypoglycemic and lipid-lowering effect, as well as immunomodulatory roles (Yu et al.). In the study “*Protective effects of polysaccharide SH-P-1-1 isolated from Phellinus igniarius on hyperuricemia and gout via intestinal microecosystem regulation*” showed that the isolated SH-P-1-1 polysaccharide from *Phellinus igniarius* was able to mitigate hyperuricemia and gout by reshaping the intestinal microecosystem (Wang et al.). Encapsulated nanoselenium-complexed *Acanthopanax* polysaccharides exhibited superior stability and antioxidant capacity in the work *Preparation and characterization of Acanthopanax polysaccharides nanoselenium with enhanced stability and antioxidant activity* (Feng et al.). Collectively, these works revealed the potentialities from polysaccharides in gut modulation, antioxidant, prebiotic, and immunomodulatory roles. Moreover, the study *In vitro fermentation characteristics and prebiotic activity of polysaccharides* demonstrates how dietary polysaccharides can modulate gut microbial communities and promote beneficial metabolic outcomes (Lai et al.), while the work *The role of polysaccharides in immune regulation through gut microbiota: mechanisms and implications* (Zhao et al.), revealed the mechanism from which polysaccharides can made the immune regulation via gut microbiota mechanisms. These researches bridges food technology and immunology for therapeutic applications.

Beyond their physiological roles, polysaccharides also offer significant opportunities for technological and industrial innovation. Due to their natural abundance, biodegradability, and biocompatibility, polysaccharides are increasingly utilized as functional ingredients in food systems. They can serve as stabilizers, thickeners, gelling agents, and encapsulation matrices for bioactive compounds, thereby improving the stability and bioavailability of nutraceuticals and functional food ingredients. Moreover, polysaccharide-based materials are being explored for applications in controlled-release delivery systems, edible coatings, biodegradable packaging, and biomedical biomaterials.

Another important dimension addressed in the Research Topic is the valorization of agricultural residues and underutilized biomass as novel sources of polysaccharides. The recovery of bioactive polysaccharides from agro-industrial by-products aligns with the principles of the circular bioeconomy, enabling the transformation of low-value biomass into high-value functional ingredients. Such approaches contribute simultaneously to sustainability goals and innovation within the food and nutraceutical sectors.

Despite significant progress in this field, several challenges remain. The structural heterogeneity and complexity of polysaccharides often complicate their standardization and reproducibility across studies. Furthermore, although numerous *in vitro* and animal studies have demonstrated promising biological activities, clinical evidence supporting the health benefits of specific polysaccharides remains limited. Future research should therefore focus on establishing standardized extraction protocols, elucidating structure–activity relationships through advanced analytical methods, and conducting well-designed human intervention studies.

In conclusion, the contributions included in this Research Topic highlight the rapidly expanding role of polysaccharides as multifunctional components in nutrition science, food technology, and biomedical research. Advances in extraction technologies, structural characterization, and functional evaluation continue to uncover new opportunities for utilizing polysaccharides to improve human health and promote sustainable food systems. Continued interdisciplinary collaboration among food scientists, chemists, microbiologists, and clinicians will be essential to fully realize the potential of these complex biomacromolecules in the development of next-generation functional foods and nutraceuticals.
